# Temporal acuity of vision decreases with eccentricity in virtual reality and is associated with schizotypy

**DOI:** 10.1038/s41598-025-03981-x

**Published:** 2025-07-01

**Authors:** Francois R. Foerster, Anne Giersch, Paola Agalliu, Axel Cleeremans

**Affiliations:** 1 Consciousness, Cognition and Computation Group (CO3), Center for Research in Cognition and Neurosciences (CRCN), ULB Neuroscience Institute (UNI), Université Libre de Bruxelles (ULB), Brussels, Belgium; 2 Department of Life Sciences, Royal Military Academy (RMA), Brussels, Belgium; 3 INSERM U1329 ‘Strasbourg Translational Neuroscience & Psychiatry’ (STEP, team Psychiatry), University of Strasbourg, University Hospital of Strasbourg, Strasbourg, France

**Keywords:** Visual temporal acuity, Temporal binding, Schizotypy, Simultaneity, Asynchrony, Schizophrenia, Perception, Personality

## Abstract

Temporal acuity reflects our ability to consciously detect a perceptual change within a short period of time, such as an asynchrony separating two visual events. In this virtual reality study, fifty participants performed a simultaneity judgment task to estimate temporal acuity across the visual field and filled the schizotypal personality questionnaire. Topographic maps were computed to visualize asynchrony discrimination skills across the visual space in two different (natural and artificial) static virtual environments. We investigate visual temporal acuity in periphery, and how estimates of temporal acuity in a psychophysical-like setting translates into a naturalistic-like scenario. First, the temporal acuity of vision decreases as the eccentricity of the targets increases, but it remains constant across meridians. Second, this deterioration of temporal coding in peripheral vision concerns non-medicated individuals self-reporting perceptual and cognitive schizotypal traits. Third, temporal acuity estimated in a traditional psychophysical visual context does not generalize to an ecologically-valid landscape scenery, such that asynchrony discrimination skills are reduced under natural vision conditions. The results suggest that distinct temporal mechanisms drive visual temporal acuity in central and peripheral vision. Furthermore, perceptual and cognitive disturbances in the neurotypical population may be linked to abnormal temporal processing in peripheral vision. Overall, these findings may pave the way toward novel investigations into the variety of time experiences across neurotypical and neurodivergent populations.

## Introduction

Visual acuity serves as a metric for a system’s ability to discriminate details, either spatially or temporally. Temporal acuity reflects one’s ability to consciously perceive change within a very short period of time. While visual spatial acuity varies across and around the visual space (reviewed in refs^1,2^), the anisotropy of visual temporal acuity remains elusive, especially in ecological settings. Distinct temporal mechanisms between central and peripheral vision^3^ could explain a disparity of temporal acuity across the visual space. Temporal processing holds particular significance in peripheral vision for detecting the sudden movement onset of incoming objects and maintaining stability in posture and perception within the environment^4,5^. Asynchronies are frequently elicited in periphery by self-motion and dynamic appearance of occluded objects. Even though self-motion helps in estimating depth and providing a sense of direction and heading^6–9^, processing asynchronies becomes complex when external objects are also moving^10,11^. The processing of asynchronies structures temporal information processing and, in turn, affects how individuals are immersed in their environment^12^. While individuals’ ability to discriminate asynchronies across the visual field remains unexplored, such research could foster significant insights regarding psychopathologies. Specifically, people in the schizophrenia spectrum disorders (SSD) exhibit difficulties discriminating asynchronies in central vision (reviewed in ref^13^) and struggle to interact with their environment^14^. This struggle may be exacerbated in peripheral vision, further impacting their ability to temporally structure their experience of the surroundings.

Over the last two decades, studies repeatedly revealed deficits of temporal acuity in patients with schizophrenia^13,15–20^, autism and dyslexia^20,21^, alongside enhancement in video game players^22^. In those studies, temporal acuity denotes one’s ability to discriminate simultaneous from asynchronous visual signals. The impairment of this ability has been related to disorganization symptoms in schizophrenia^15,23^. Not being able to structure events in time at the level of milliseconds may represent a basic impairment impeding patients from organizing and making sense of their environment. In line with these studies, the temporal integration window enabling to segment information in time appears to be altered in SSD^15,24–26^ and schizotypy^27–29^. It is striking that patients themselves report visual organization disorders when they walk, that is when they need to process movements and the succession of events in the periphery. A patient reports that ‘Moving is like a motion picture. If you move, the picture in front of you changes. The rate of change in the picture depends on the speed of walking. If you run you receive the signals at a faster rate. The picture I see is literally made up of hundreds of pieces.’ Another patient says that ‘Everything is all right when I stop. If I move everything I see keeps changing, everything I’m looking at gets broken up and I stop to put it together again.’^30^. More recently, Schultze-Lutter et al. described basic symptoms in schizophrenia^31^, including abnormal perceptual experiences, that may help early detection of psychosis. However, as emphasized by the authors, those perceptual impairments descriptions may not be robust enough to be used in clinical practice^32^. An experimental approach may help to better understand those impairments and to enable their use in clinical settings.

Some proposed that abnormal perceptual experience found in schizophrenia rely on spatiotemporal processing deficits^23,33–35^, but those deficits were observed in foveal vision. Interestingly, some authors attributed cognitive alterations in schizophrenia to attentional processing deficits in peripheral vision^36,37^. Yet, how patients process spatiotemporal information in peripheral vision remains unclear. Previous simultaneity judgments tasks also evidenced unisensory and multisensory temporal acuity deficits in individuals presenting high schizotypal personality traits^19,25,27–29,38–40^, thus highlighting temporal processing alterations across the schizophrenia spectrum and schizotypy. Schizotypy is seen as belonging to a continuum between personality disorders and schizophrenia^41^. Identifying which perceptual capabilities are impaired or preserved in individuals with schizotypal personality traits thus informs on difficulties in SSD^42^. Moreover, given the fact that individuals with schizotypy are prone to develop schizophrenia^43^, their difficulties might represent risk factors for SSD while ruling out the impact of long-term medical treatments on task performance (see^44^). Given the self-reported perceptual disturbances in the schizophrenia spectrum^30,31,45^, a possibility is that their deficits of temporal acuity not only occur in the fovea but are also present in peripheral vision, where temporal processing is crucial in maintaining perceptual stability. Understanding the spatial characteristics of visual temporal acuity across the general population could help refining temporal acuity measurements as a marker of personality disorders, along with interventions targeting spatiotemporal visual processing disorders.

The primary goal of this study is to evaluate the putative uniformity of the temporal acuity across and around the binocular visual field. Prior research has yielded inconsistent findings regarding the anisotropy of temporal vision, especially the idea of heightened temporal acuity in peripheral fields. On the one hand, temporal acuity appears to improve with eccentricity. For instance, both the ability to detect a flickering stimulus^46,47^ (i.e., the critical flicker frequency) and the speed of information processing^48,49^ have been described as increasing with greater eccentricity. In contrast, other studies have suggested that temporal acuity declines with eccentricity. For instance, the ability to detect a temporal gap between two pulses of light has been described to monotonically decrease from 5° to 20° of visual angle^50,51^ and the temporal acuity to saturate at 10 Hz in non-foveal vision^3^. Moreover, motion detection skills decline with greater eccentricities^52,53^ and both horizontal–vertical anisotropy^54–56^ and vertical meridian asymmetry^57^ were found in numerous aspects of motion processing, suggesting that spatiotemporal visual acuity varies around the visual field (but see ref^58^ for more recent contrasting findings). Inter-individual differences may explain these discrepancies, mostly found in the psychophysics literature. It is important to note that these discrepancies may also stem from the use of tasks involving rapid serial visual presentation of multiple stimuli at a single location, which could introduce masking confounds altering temporal acuity estimations^59^. Tasks that involve visual simultaneity judgments (SJ) make it possible to estimate the temporal acuity without masking confounds. This study aims to evaluate the hypothesis (H_1_) that visual temporal acuity varies across and around the visual field. Crucially, we extended investigations suggesting that temporal acuity may represent a biomarker of both schizophrenia and schizotypy^15,23,32^ by measuring schizotypal traits in participants.

This study leverages signal detection theory and virtual reality methods to evaluate the anisotropy of visual temporal acuity across neurotypical individuals reporting perceptual and cognitive symptoms of schizotypy. It remains unclear whether temporal acuity deficits can be attributed to attentional processing difficulties, since attention deficits are observed in SSD^60^ and schizotypy^61,62^, especially across the visual space^36^ and under high perceptual load^63^. The role of perceptual load questions the validity of tests of temporal acuity built in laboratories, relative to real-world contexts, and this is especially important when evaluating neurodivergent individuals. Indeed, the visual processing of complex but not simple images appears to be altered in schizophrenia patients^64^. This led to our assumption that temporal acuity deficits found in individuals with schizotypal traits may not only be accrued in peripheral vision (hypothesis H_2_), but also during complex visual processing (hypothesis H_3_) due to extensive attentional resources required to perform visual tasks, as in real-life situations. Recent work indeed suggests that some timing mechanisms depend on the visual context. For example motor timing is more precise while being exposed in natural landscapes rather than urban environments^65^. As far as we know, it remains unknown how estimates of visual temporal acuity translate from psychophysical settings to naturalistic settings, especially given the disparity in low-level features and perceptual contrast across space in ecological scenarios. To test this hypothesis, participants performed the SJ task in two different immersive virtual environments, namely a minimal environment (involving a grey background and no perceptual load, like in traditional psychophysical setups) and a complex natural environment (including a landscape background with heterogenous luminance, visual features and high perceptual load, typical of real-life situations).

## Methods

### Participants

The sample size was estimated using G*Power (v. 3.1.9.6)^66^. Fifty participants were sufficient to reach above 99% of statistical power at α = 0.05 for a medium effect size (η^2^_p_ = 0.25) concerning the main effects of the experimental conditions on perceptual sensitivity, using ANOVAs. Therefore, fifty adult volunteers were recruited in this study. All participants reported having normal or corrected-to-normal vision. Four participants were discarded, one due to severe drowsiness during the task, one due to diplopia, one due to cyber-sickness symptoms, and one due to task performance at chance level. No other participant reported any feeling of cyber-sickness during or after the experiment. The final sample was composed of 46 participants (mean age = 19.9, range 17–52 years old, including 39 women and one left-handed). Eye-tracking data from two participants was discarded because of ineffective recording due to the wearing of glasses. All participants were naïve to the purpose of this study and provided written informed consent before participating. The study was approved by the local ethics committee of the Université Libre de Bruxelles and the experimental procedure was in agreement with the Helsinki declaration.

**Fig. 1:**
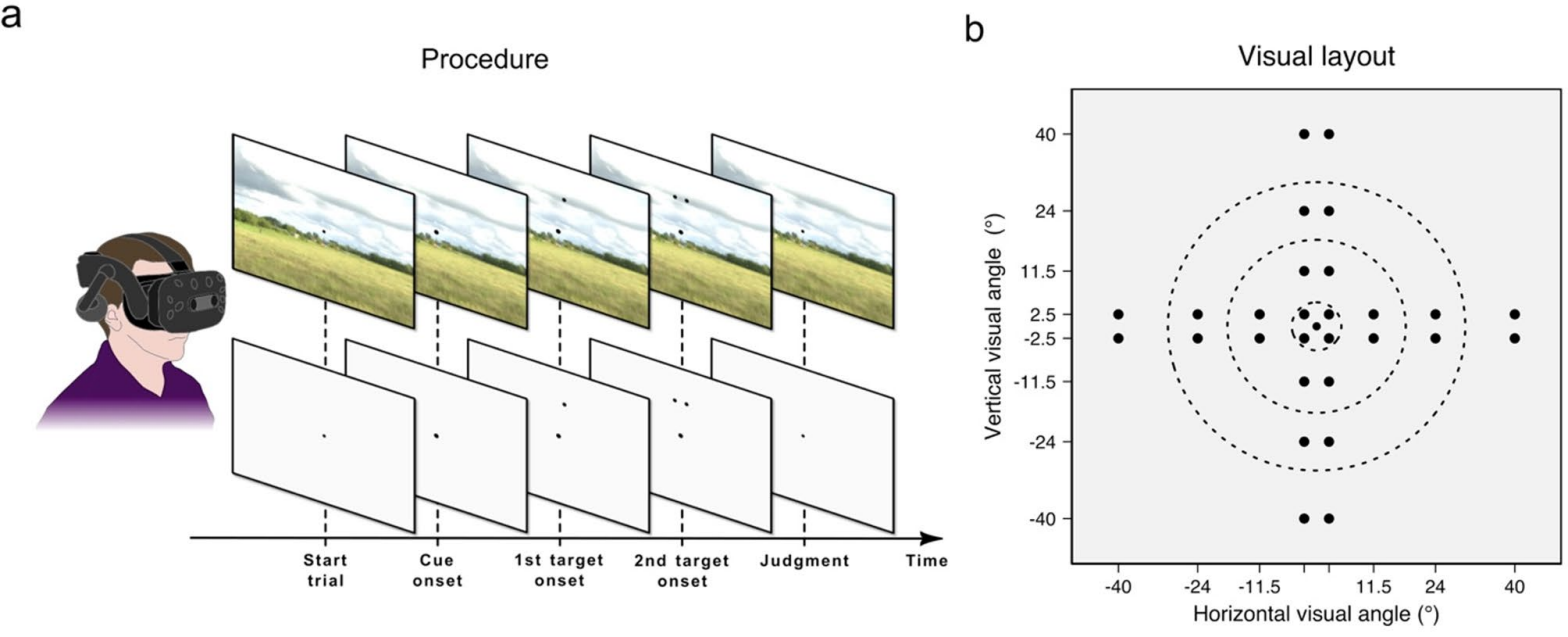
Experimental task and stimulus layout. (**a**) Participants performed a simultaneity judgment task consisting of discriminating synchronous (0 ms SOA) from asynchronous (22 ms SOA) onsets of two visual targets (black spheres). Participants performed the task in both ecologically-valid (top) and minimal virtual environment (bottom). (**b**) Layout of the possible locations of target presentation. The pair of targets were presented at different possible eccentricities from a central gaze fixation point and within different possible polar angles.

### Experimental setup

 The virtual environments (VR) were developed with the Unity software (Unity Technologies, v. 2019.3.9f1). The HTC Vive Pro Eye (HTC Corp.) headset and controllers immersed participants in VR. Participants wore the VR headset while sitting on a comfortable chair. In this study, participants were immersed either in a minimal environment (an empty grey space, Fig. 1a) or in a natural 360° panoramic picture of a landscape (Fig. 1b). This natural landscape included tall grass and a cloudy sky, with houses and trees on the horizon.

The task used a visual layout that included a gaze fixation point and 16 possible locations for pairs of stimuli (hereafter called targets). All stimuli were black spheres. Each pair of targets was displayed at different eccentricities (i.e., 2.5°, 11.5°, 24°, and 40° of visual angle from the fixation point) and in one of the four polar angles (i.e., upper, lower, left, and right polar angles). The distance separating two targets within each pair was of 5° of visual angle. The location of all stimuli was contingent to the head orientation, resulting in a constant distance separating the participants and all stimuli. The constant perceived size of the targets was of 1.15° of visual angle. The SRanipal SDK (v. 1.1.0.1) allowed monitoring eye blinks occurring around the onset of the targets.

### Task

Participants were instructed to discriminate simultaneous from asynchronous onsets of the two targets. During the task, participants continuously focalized their gaze on the fixation point. At the start of each trial, the fixation point increased in size (from 0.57 to 0.71° of visual angle), to warn participants about the upcoming appearance of a pair of targets presented within a random time interval varying between 1000 ms and 2000 ms. Next, a pair of targets was presented with either simultaneous (0 ms SOA) or asynchronous (22.2 ms, i.e., 45 Hz) onsets. The targets remained visible until the participants’ response so as to avoid apparent motion that can result from their offset^67^. Participants answered by pressing the trigger button or the pad button of the VR controller placed in their right hand. The matching of the controller buttons with the judgments (simultaneous vs. asynchronous) was randomly assigned for each participant. No response time limit was implemented. Upon participants’ response the targets disappeared and the fixation point returned to its initial size (0.57° of visual angle) after 1000 ms.

The training phase comprised 20 trials with a preselected set of targets presented at different eccentricities and polar angles. The test phase consisted of three trial blocks for each environment, for a total of six trial blocks and 384 trials lasting approximately 40 min. Each trial block included four pairs of targets presented at each of the four eccentricities and polar angles, resulting in 64 trials per trial block. Among these four pairs of targets, two were presented simultaneously and two were presented asynchronously. Thus, half of the pairs of targets were presented simultaneously while the other half were presented asynchronously, leading to a 50% chance of correct judgment at random. Participants performed 12 trials for each eccentricity, polar angle and environment.

The virtual environment alternated between each trial block. In both virtual environments, participants were instructed to maintain the head and body posture straight, but also to keep the fixation point above the horizon in the natural environment. These instructions were implemented to keep the location of the targets constant across participants and virtual environments. These locations were verified visually on-screen by the experimenter throughout the task. The order of presentation of the environments was counterbalanced across participants. At the beginning of each trial block, the orientation of the field of view was set randomly (from 0° to 360°). This allowed each participant to perform the natural environment task in a novel orientation on each trial block. Participants were proposed to remove the headset between each trial block for a break. Trials containing an eye blink during the presentation of the targets (time-window starting from 0 ms to + 100 ms from the first target presentation) were discarded (*N* = 146 or 0.9% of the trials) to ensure efficient target processing.

### Data acquisition and analysis

The eye-tracking system (Tobii Ltd.) integrated into the VR headset monitored the binocular gaze position at a sampling rate of 90 Hz and was used to extract eye blinks. The eye tracker was calibrated at the start of the experiment and following each removal of the headset.

Signal detection theory was applied to the behavioural data to distinguish one’s ability to discriminate between simultaneous and asynchronous targets (namely, the sensitivity *d’* value) from one’s decision bias (also called criterion *c* value, which is here the tendency to report perceiving simultaneous onsets of the targets independently of the presence or absence of the SOA, see Suppl. Mat. for details). The limited number of SOAs does not allow us to estimate the visual temporal integration window but rather the ability to differentiate between the two specific SOAs (0 ms vs. 22 ms).

### Schizotypal personality questionnaire

Participants were asked to complete the Schizotypal Personality Questionnaire (SPQ)^68^ presented in a paper and pencil version during breaks. The SPQ is a commonly used self-report questionnaire estimating the presence of schizotypal traits. The questionnaire encompasses 74 questions with “Yes’’ or “No” possible answers concerning different aspects of the personality, sensorial experiences and beliefs. The questionnaire contains nine subscales divided into a three-components model: Cognitive-Perceptual (subscales: ideas of reference, unusual perceptual experiences, magical thinking, suspiciousness), Disorganization (subscales: odd speech, odd or eccentric behavior) and Interpersonal (subscales: excessive social anxiety, suspiciousness, no close friends, constricted affect) scales. We focus on the cognitive-perceptual subscale given the assumption that altered temporal acuity may impact perceptual experiences.

### Statistical analysis

R (v.4.3)^69^ and the rstatix (v. 0.6.0) package were used to perform two-sided repeated-measures analyses of variances (rANOVAs). All rANOVAs were performed with a Greenhouse-Geisser correction when the within-subject factors violated the sphericity assumption.

Planned comparisons were performed with Bayesian *t*-tests (BayesFactor package)^70^ and False-Discovery Rate (FDR) *p*-value correction^71^. All Bayes Factors (BF) were calculated using a standard Cauchy prior (*s* = 0.707) for the alternative hypothesis. Reported BF_10_ values indicate how many times more evidence there is in favor of the alternative hypothesis compared with the null hypothesis. Conversely, BF_01_ values indicate how many times more evidence there is in favor of the null hypothesis compared with the alternative hypothesis. The absence of main effects in the rANOVAs was ascertained using Bayesian linear regressions (lmBF() from the BayesFactor library) comparing models with and without the variable of interest. In these control analyses BF_01_ were estimated using 10^6^ Markov chain Monte Carlo simulations.

## Results

### Temporal acuity varies across but not around the visual space

**Fig. 2:**
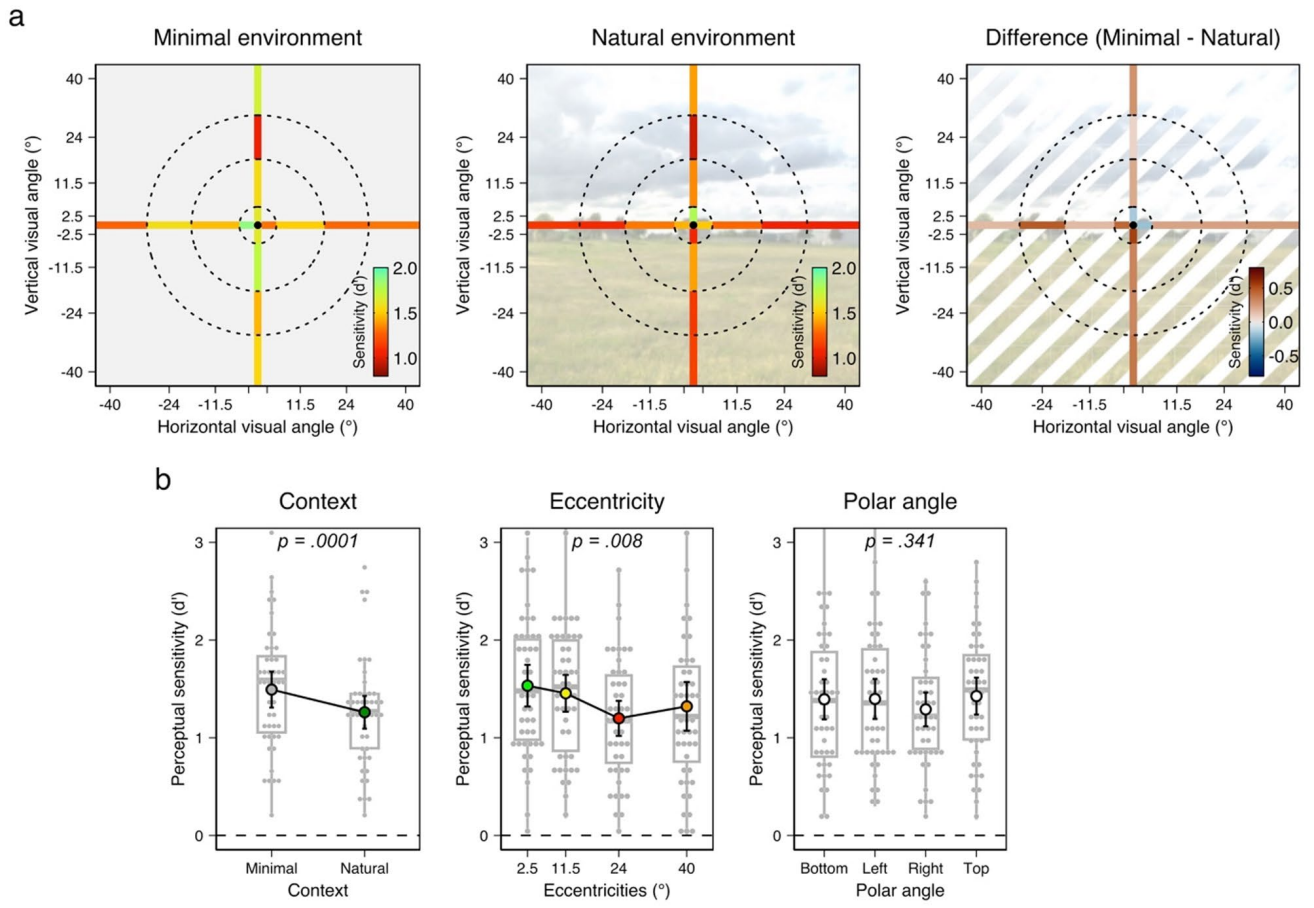
Visual temporal acuity varies across visual context and eccentricity, but not across the polar angles of targets presentation. (**a**) Topographic maps of sensitivity to 22 ms asynchronies in the minimal environment (left), natural environment (middle), along with the difference between the two static virtual environments (right). The concentric circles represent the different areas of visual stimulation and were not presented during the task. (**b**) The sensitivity to asynchronies was reduced in the natural environment (left) and in peripheral vision (middle), but remained constant across the polar angles (right). Error bars represent a 95% confidence interval from the mean.

First, a preliminary analysis verified participants’ sensitivity to the 22 ms asynchrony. For each participant, a one-tailed *t*-test against zero evaluated their perceptual sensitivity (*d’*). Each *t*-test involved 32 observations (two contexts, four eccentricities, and four polar angles). This procedure revealed that all participants were able to discriminate simultaneous from asynchronous stimulus onset (all *p* < 0.001) except one (*d’*_mean_ = 0.14, *p* = 0.19) who was thus discarded (as mentioned above). Second, we evaluated participants’ ability to discriminate the asynchrony across the virtual environments, as well as across and around the visual field (Fig. 2a). A three-way rANOVA with the factors Context, Eccentricity, and Polar angle was applied to perceptual sensitivity measures (*d’* values) and showed a main effect of Context (*F*(1,45) = 17.89, *p* = 0.0001, *η*^*2*^_*p*_ = 0.284; Fig. 2b), such that the participants’ sensitivity to the asynchrony was significantly lower in the natural (Mean = 1.26, SD = 0.57) than in the minimal environment (Mean = 1.49, SD = 0.62, Cohen’s *d* = 0.624, CI_95%_ = [0.34, 1]). In line with this result, a complementary analysis revealed both a lower judgment accuracy and longer decision times in the natural environment compared with the minimal environment (see details in Suppl. Mat, Fig. S1 & Fig. S2). The rANOVA analysis also reported a main effect of the Eccentricity (*F*(3,135) = 4.136, *p* = 0.008, *η*^*2*^_*p*_ = 0.084). Planned comparisons revealed that perceptual sensitivity is significantly higher when stimuli are presented at 2.5° of visual angle rather than at 24° (two-sided *t*-test _FDR−corrected_, *t*(46) = 3.3, *p* = 0.011, Cohen’s *d* = 0.49, BF_10_ = 16.6) and when presented at 11.5° of visual angle rather than at 24° (two-sided *t*-test _FDR−corrected_, *t*(46) = 4.19, Cohen’s *d* = 0.62, *p* = 0.0001, BF_10_ = 188). The sensitivity appears similar between 2.5° and 11.5° (BF_01_ = 4.4, Cohen’s *d* = 0.13) and between the 24° and 40° (BF_01_ = 3.19, Cohen’s *d* = 0.17). In line with this result, lower judgment accuracy and longer decision times were reported when the eccentricity of the stimuli increase (see details in Suppl. Mat., Fig. S1 & Fig. S2). The rANOVA reported no main effect of the Polar angle (*F*(3, 135) = 1.126, *p* = 0.34, *η*^*2*^_*p*_ = 0.084). A Bayesian linear model comparison strongly suggests the absence of effect of the Polar angle on the perceptual sensitivity (BF_01_ = 160). Finally, the rANOVA reported an interaction effect between the Eccentricity and the Polar angle (*F*(9, 405) = 2.128, *p* = 0.026, *η*^*2*^_*p*_ = 0.045). In the upper visual field (and not in the other parts of the visual field), sensitivity to asynchrony gradually decreases from 2.5° to 24° of eccentricity but remains comparable between 2.5° and 40°. This observation was confirmed within both minimal and natural environments. However, the low statistical power associated with this unexpected interaction effect (44.6%) prevents drawing robust conclusions. No other interaction effect was found (all *F* < 1.87, all *p* > 0.137). Another Bayesian linear model comparison strongly suggests the absence of interaction effect between the Context and the Eccentricity on the perceptual sensitivity (BF_01_ = 172). In summary, the perceptual sensitivity to the 22 ms asynchrony decreases in the most natural visual scenery, but also decreases as the eccentricity of the stimuli increases.

A control analysis revealed Bayesian evidence for an absence of decision bias (*c* criterion) across the Setting, the Eccentricity and the Polar angle factors (see details in Suppl. Mat., Fig. S3). No evidence for an effect of gender nor age on sensitivity was found. Decision bias was higher in man than women but did not vary across age (see details in Suppl. Mat.). Raw data (hit rate, false alarm rate, and percentage of ‘asynchronous’ response) are provided in Suppl. Mat (see Fig. S4).

### Schizotypal traits are associated with reduced Temporal acuity of vision

Previous studies suggested that temporal processing is affected in the presence of schizotypal personality traits. Here, we sought to evaluate whether this alteration can predict the gradient decline of temporal visual acuity towards the peripheral visual fields. As in previous studies^25,27,40,42,72^, we focused on the cognitive-perceptual subscale of the SPQ.

**Fig. 3:**
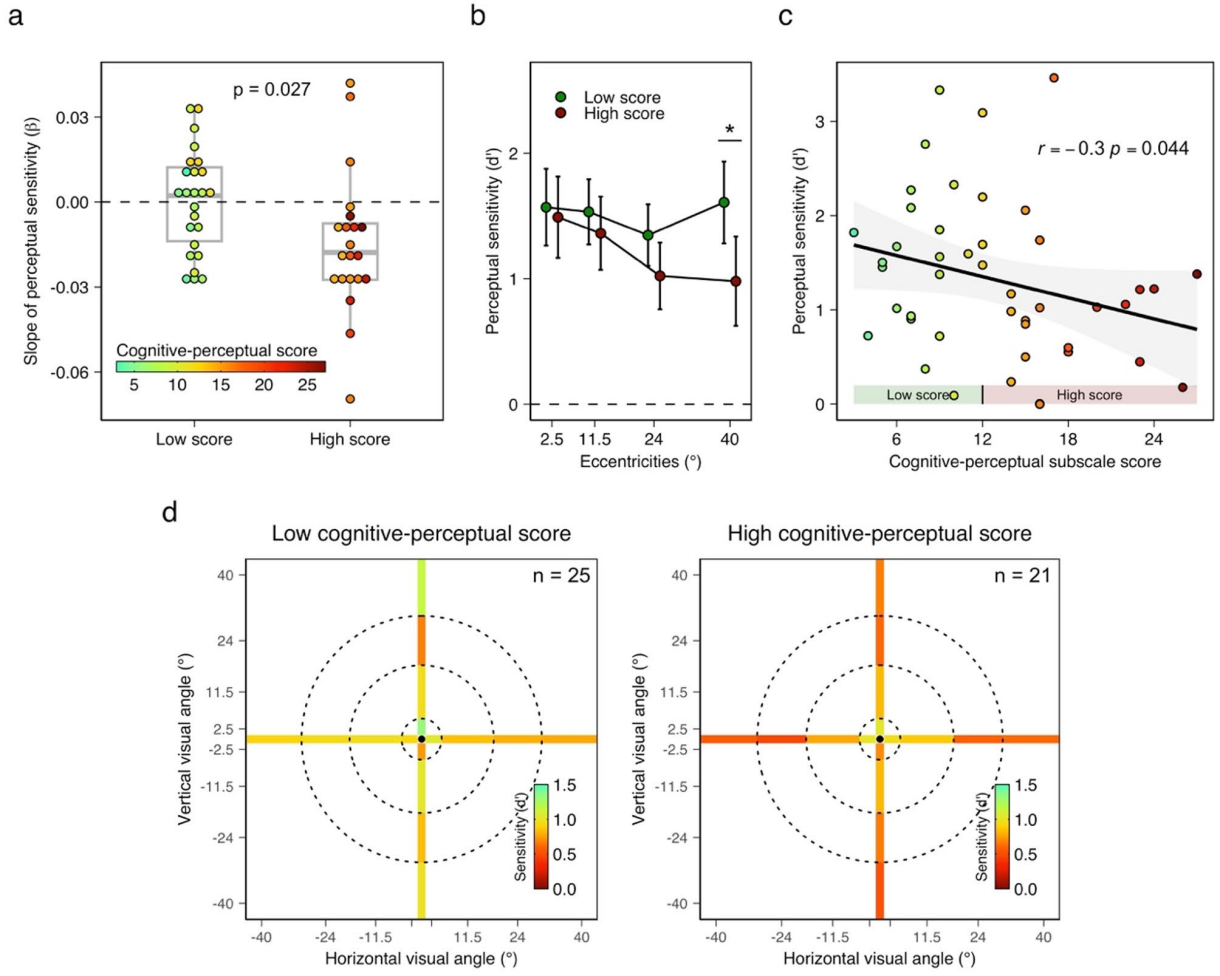
Schizotypal personality traits are associated with visual temporal acuity alterations across space. (**a**) A linear trend reflecting the gradual decline of sensitivity to asynchronies across eccentricities was found in individuals with a high cognitive-perceptual score. (**b**) The sensitivity to asynchronies was reduced in peripheral vision (40° of visual angle) in individuals with a high cognitive-perceptual score. (**c**) The cognitive-perceptual score predicts the sensitivity to asynchronies in peripheral vision (40° of visual angle). (**d**) Topographic maps of sensitivity to asynchronies in individuals with low (left) and high (right) cognitive-perceptual scores. Error bars represent a 95% confidence interval from the mean. * *p* < 0.05.

First, we separated participants into two groups depending on whether their scores were below (N _Low score_ = 25) or above (N _High score_ = 21) the median score across participants (median = 12). No age (two-tailed *t*-test, *p* = 0.33, BF_01_ = 2.2) nor gender (Chi^2^ test, *p* = 0.42) difference was found between groups. Then, for each participant and each environment, we fitted a linear regression on the perceptual sensitivity (*d’*) values estimated across the four eccentricities. Negative values of the slope (β coefficients) indicate a decrease of temporal perceptual sensitivity as the eccentricity of the stimuli increases. A two-way rANOVA applied to these slope coefficients of perceptual sensitivity revealed a significant effect of the between-subject categorical factor Cognitive-perceptual score (*F*(1,44) = 5.265, *p* = 0.027, η^2^_p_ = 0.107, BF_10_ = 2.32), such as participants with a high score have a steeper negative slope (β _Mean_ = -0.0145) than participants with a low score (β_Mean_ = 0.0001, Cohen’s *d* = 0.67, CI_95%_ = [0.06, 1.45], see Fig. 3a). Contrasting with our hypothesis, no effect of the Setting (*F*(1,44) = 0.40, *p* = 0.53, BF_01_ = 4.06) nor interaction effect (*F*(1,44) = 0.55, *p* = 0.46, BF_01_ = 2.99) was revealed. One-tailed t-tests against zero indicated that the slopes are significantly lower than zero in participants with high cognitive-perceptual scores (*t*(20) = -2.67, all *p* = 0.007, BF_10_ = 3.69, Cohen’s *d* = 0.58, CI_95%_ = [-1.36, -0.14]) but do not differ from zero in participants with low score (*t*(24) = 0.03, all *p* = 0.51, BF_01_ = 4.74).

This effect could be due to a group difference in the sensitivity in either the near-foveal or the peripheral visual fields. To distinguish between these two possibilities, we applied an rANOVA to the perceptual sensitivity values with the factors Cognitive-perceptual score and Eccentricity (Fig. 3b). The analysis revealed an interaction effect (*F*(3,132) = 2.845, *p* = 0.04, *η*^*2*^_*p*_ = 0.061, BF_10_ = 1.66), such as the perceptual sensitivity decreased in participants with a high cognitive-perceptual score when the stimuli were presented at 40° of visual angle (two-sided *t*-test _FDR−corrected_, *t*(42.8) = 2.701, *p* = 0.039, BF_10_ = 4.95, Cohen’s *d* = 0.799, CI_95%_ = [0.23, 1.65]), but not when the stimuli were presented at other eccentricities (all *p* > 0.27, all BF_01_ > 0.85). A Spearman correlation confirms this result by depicting a negative association between the perceptual sensitivity to asynchronies presented at 40° of visual angle and the cognitive-perceptual score (*r* = -0.3, *p* = 0.044, see Fig. 3c). In other words, the deterioration of temporal acuity as the eccentricity of the stimuli increases mainly concerned participants self-reporting cognitive-perceptual traits of schizotypy (see Fig. 3d). Spearman correlations do not suggest any relationship between the overall sensitivity to asynchronies and model factors or subscales except the “Odd beliefs or magical thinking” subscale (*r* = -0.31, *p*_*uncorrected*_ = 0.039, BF_10_ = 2.8; see Fig. S5). This correlation strengthens the link between these results and schizotypy.

## Discussion

Results regarding the existence of anisotropy in temporal processing are inconsistent^46,47,50,51,73^ although Yo & Wilson proposed a distinction between temporal mechanisms in central and peripheral vision^3^. The present study capitalized on immersive virtual reality to evaluate the hypothesis of a heterogeneous distribution of temporal acuity within the visual space. Participants performed a simultaneity judgement task consisting in judging whether two targets were presented simultaneously or asynchronously. Our results are twofold. First, at the group level, the temporal acuity of vision declines with eccentricity, indicating a non-uniform sensitivity to small asynchronies across the visual space. Second, the results revealed that the gradual loss of visual temporal acuity with eccentricity is predominant in non-medicated individuals self-reporting schizotypy symptoms.

We found evidence consistent with our first hypothesis (H_1_) that the temporal acuity decreases with visual eccentricity. Our results shed new light upon longstanding conflicting results concerning the spatial distribution of visual temporal acuity. While previous studies using critical flicker frequency paradigms reported an increase in temporal acuity with eccentricity^46,47^, those employing double pulse techniques indicated a decrease^50,51^. More recent evidence and arguments can explain these discrepancies. During rapid serial visual presentation at a single location, forward and backward masking effects alter visual processing^59,74^. Consequently, both critical flicker frequency and double pulse paradigms might suffer from masking confounds that bias estimations of temporal acuity. Such paradigms may thus not reflect the temporal discrimination skills used in many ecological situations in which stimuli are apart in space (e.g., perceiving the asynchrony between two athletes, dancers, or cars). Most importantly, our analysis highlights that the decline of temporal acuity with eccentricity is not universally observed in neurotypical individuals. Here, targets were located at the center of distinct visual processing areas, namely within foveal (2.5° of visual angle) and macular (11.5° of visual angle), near peripheral (24° of visual angle) and mid peripheral (40° of visual angle) vision. The reduced discrimination skills beyond macular visual processing predominantly concerned individuals reporting perceptual and cognitive disturbances. This highlights a potential influence of sample biases in previously reported inconsistent results concerning the anisotropy of temporal processing – visual temporal acuity varies across space in some but not others. It is indeed important to note that we did not select our participants on the basis of their scores at the SPQ. The scores on the SPQ only reflect the usual inter-individual differences found in the general population, and may thus have affected previous results in the literature.

In accordance with our second hypothesis (H_2_), the observed deterioration of temporal acuity in peripheral vision predominantly concerned individuals reporting abnormal perceptions belonging to schizotypy symptoms in daily life^68^, such as the feeling of being watched or hearing their thoughts aloud. A recent series of studies show that temporal processing deficits are present across the schizophrenia spectrum, and several suggest that the deficit might be larger for multisensory information^19,25,27,28,38,39,75^. However, the presence of deficits in temporal acuity in visual unisensory conditions in schizophrenia^15–18,24,26,76,77^ along with the present deficit in the periphery in schizotypy suggest that the ability to structure information from peripheral visual inputs in time might be crucial to the symptomatology of SSD. A recent theoretical proposal postulates that SSD symptomatology could rely on mis-alignments in timing mechanisms at unconscious and conscious levels^78,79^. Our results complement this literature in revealing an impaired timing mechanism in neurotypical individuals presenting symptoms of SSD. These results showing accrued unisensory temporal acuity deficits in peripheral vision questions how individuals interact with their environment when having to structure information in time.

Several possibilities could explain the present temporal deficit in peripheral vision in individuals with schizotypal traits. From a cognitive point of view, a longstanding line of research demonstrated that attentional dysfunction, including sustained attention^80,81^, divided attention^36^, attentional capturing^61^ and hyperfocusing^37,62^ partly explain cognitive deficits found in SSD. However, attentional dysfunction alone may not fully account for the observed temporal deficits. First, we found preserved discrimination skills within the range of eccentricities used in these attention studies, which are typically involving macular vision. Second, the size of the attentional spotlight may not affect temporal acuity^82^. Third, contrasting with our results, the hyperfocusing hypothesis would have predicted enhanced acuity in foveal vision in individuals presenting schizotypal traits. Fourth, by assuming that enhanced attention is required to perform the task in the natural setting, our lack of an interaction effect between the environmental settings and groups of individuals on asynchrony discrimination does not support this explanation of attentional dysfunction. Indeed, we anticipated an exacerbation of temporal acuity deficit in individuals with schizotypal traits in the natural setting. Yet, our analysis of sensitivity across virtual environments contrast with this hypothesis (H_3_). Consequently, it appears unlikely that the temporal deficits found in individuals with schizotypal traits can be attributed to altered attentional processing. Instead, temporal deficits in individuals with high schizotypy scores may rely on alterations in sensory processing rather than dysfunctions at higher cognitive levels. It might be tempting to attribute these effects to coding in the magnocellular pathway, given the sensitivity of this pathway to temporal transients. However, it has recently been shown that the proportion of parvo- and magnocellular cells is uniform across the retina in humans^83^. This possibility would thus have to be explored further, especially since detecting an asynchrony is not only about coding a single unique transient, but mainly about comparing the temporal onset between distinct signals. In other words, processing visual asynchronies across the visual space is about structuring information in time.

Thus, on the one hand, peripheral vision is crucial in everyday life to monitor our surroundings, detect new incoming moving objects^84^ and adapt our locomotion and visual search accordingly (reviewed in ref^85^). On the other hand, the processing of asynchronies shapes how information is integrated or segregated in time^86^, which is particularly critical during locomotion and visual search. Thus, processing asynchronies becomes crucial in peripheral vision to structure sensory events in time and adapt our behaviors in highly dynamic environments. In turn, detecting and comparing such temporal changes across peripheral and central vision can help generate a coherent representation of the world^87^ and facilitate acting within it. The fact that subjects with schizotypy symptoms show perceptual alterations in periphery but not in central vision is certainly an argument suggesting that processing asynchronies in central and peripheral vision differ^3^. Yielding different temporal mechanisms in central and peripheral vision could help maintaining a feeling of time continuity between saccades, akin to visual coherence across saccade (also called trans-saccadic integration)^87^. Indeed, it is important to note that asynchronies may also be a source of environmental instability^88^, and ignoring peripheral asynchronies might be an attempt at preserving the environment stability for individuals with abnormal perceptions. A conceivable hypothesis is that the frequency of brain oscillations in the alpha range may account for the sensitivity to visual asynchronies, as previously found in two-flashes illusion tasks (e.g^29^) and temporal order tasks^89^ (but see^22,90^ for contrasting results). Previous works report slower alpha oscillations in resting-state of patients with schizophrenia^91–93^ and neurotypical individuals presenting schizotypal traits^94^. A recent work also suggested a lack of desynchronization in the alpha-band in patients with SSD performing a simultaneity/asynchrony discrimination task. Further investigations are required in SSD and schizotypy to evaluate whether the heterogeneity of temporal acuity across the visual field may rely on these oscillatory mechanisms. Altogether, our results highlight a deficient temporal mechanism specific to peripheral vision in individuals with schizotypal traits, which both fit with the cognitive dysmetria hypothesis of schizophrenia^95^ and a recent related proposal^78,79^, but also extend them to schizotypal personality traits.

### Limitations

We acknowledge several limitations in the current study. First, it should be reminded that this study evaluates one’s ability to discriminate between two specific stimulus onset asynchronies (0 ms vs. 22 ms) across space rather than the temporal integration window. Further studies should investigate whether the visual temporal integration window is similarly sensitive to eccentricity but not polar angles. While the SJ task requires both spatial and temporal processing, it focuses specifically on discriminating temporal asynchronies. One may wonder about the extend our target presentation involves motion processing. A previous study indicates that it is unlikely that participants report seeing apparent motion when two stimuli separated by 6° of visual angle are presented with an SOA below 50 ms – meaning that a larger SOA is required for apparent motion at this distance (see ref^96,97^ for a review). Here, participants judged targets’ onset as ‘simultaneous’ rather than ‘asynchronous’ most of the time (Fig. S4), preventing the possibility for apparent motion. However, a control task devoid of temporal processing would be necessary to ensure that any observed perceptual deficit is specific to the temporal domain rather than a general deficit in reporting percepts in peripheral vision among individuals with schizotypal traits. Additionally, the fixed size of the targets hinders our ability to determine whether the gradual decline in temporal acuity with eccentricity arises from qualitative differences (i.e., different mechanisms) or quantitative differences (e.g. size and density of ganglion-cell receptive fields) in sensory processing. Further studies should vary the size of the targets to examine the role of cortical magnification (i.e. the amount of cortical areas allocated to process information within a portion of the visual space) in temporal acuity, although previous results suggest that critical flicker frequency is not affected by varying stimulus size^47^. Finally, the holistic approach embraced in our study refrains to draw conclusions upon what low-level visual features lead to the reduced visual temporal acuity found in our ecologically-valid ‘natural’ virtual environment. Further examinations are required to clarify whether specific factors or the synergy of multiple factors (e.g. luminance, perceptual contrast, clutter) limit the temporal resolution of vision in complex visual sceneries.

## Conclusion

Our study demonstrates that visual context significantly influences the temporal acuity of vision – one’s ability to detect asynchronies in artificial minimalist scenery does not generalize to ecologically-valid environments. Crucially, our results provide evidence for an inherent anisotropy of visual temporal acuity, indicating that our ability to structure events in time depends on where we are looking at. This highlights the role of spatial properties of sensory processing in shaping time consciousness and the “perceptual moment”, which refers to the window of simultaneity. Finally, we found temporal acuity to be poorer in peripheral vision in non-medicated individuals with schizotypal traits. This highlights the fact that cognitive and perceptual disturbances found in the neurotypical population are associated with an altered temporal mechanism leading one to integrate rather than to segregate information in time in peripheral vision. Given the similarity of these results with those found in central vision in individuals with schizophrenia and schizotypy, the results reinforce the concept of a continuum in basic symptoms underlying SSD across the population. The alteration observed here is particularly significant given the role played by peripheral vision to generate a coherent experience of the world and the fact that integration and segregation processes in peripheral vision are vital to adapt our behaviors in dynamic environments.

## Electronic supplementary material

Below is the link to the electronic supplementary material.

Supplementary Material 1

## Data Availability

Scripts and data are accessible via an Open Science Framework repository (https://osf.io/9swgu/).
