# Supplementary material for: Temporal acuity of vision decreases with eccentricity in virtual reality and is associated with schizotypy
Source: Sci Rep. 2025 Jul 1;15:22070. doi: 10.1038/s41598-025-03981-x (PMC12214604; doi:10.1038/s41598-025-03981-x)
Supplement: Supplementary file 1 — Supplementary Material 1 [file 41598_2025_3981_MOESM1_ESM.docx]

**Article: Temporal acuity of vision decreases with eccentricity in virtual reality and is associated with schizotypy**

**Supplemental Materials**

**Methods**

Signal detection theory is traditionally applied to stimulus detection task data using present/absent targets such as a Gabor patch which orientation may change (clockwise vs counterclockwise). There, the orientation of the Gabor patch is often disregarded. When using a SJ task (also called asynchrony detection task), signal detection theory is applied to evaluate one’s ability to distinguish asynchronous stimuli (i.e., the weak signal variable) from simultaneous stimuli (i.e., the noise variable). The presence of a SOA generates an order of presentation (e.g., left-then-right stimulus, top-then-bottom stimulus). Here, we ignored the order of the stimulus presentation. We used signal detection theory to differentiate the participants’ ability to discriminate asynchronous from simultaneous targets (i.e., the sensitivity to the asynchrony) and the participants’ decision bias (also called criterion, which is here the tendency to report perceiving simultaneous onsets of the targets independently of the presence or absence of the SOA). These sensitivity and response bias estimates are uncorrelated only under the assumption of equal distribution of signal and noise^1^. Because such distribution assumption cannot be verified in our SJ task, both distribution-dependent^1^ and distribution-independent^2^ measures of sensitivity (*d’*, *A_ROC_*) and response bias (*c*, *B_ROC_*) were estimated using the following equations:

|  | $\boldsymbol{d'=z}\left( \boldsymbol{HR} \right)\boldsymbol{-z}\left( \boldsymbol{FAR} \right)$ | (1) |
| --- | --- | --- |
|  | $c=\frac{1}{2} (z\left( HR \right)+z\left( FAR \right))$ | (2) |

and

|  | $\boldsymbol{A}_{\boldsymbol{ROC}}\boldsymbol{=}\boldsymbol{K}_{\boldsymbol{A}}\boldsymbol{+}\boldsymbol{K}_{\boldsymbol{B}}\boldsymbol{+0.5}$ | (3) |
| --- | --- | --- |
|  | $B_{ROC}=K_{A}/K_{B}$ | (4) |

given

|  | $\boldsymbol{K}_{\boldsymbol{A}}\boldsymbol{=}\frac{\boldsymbol{1}}{\boldsymbol{4}}\left( \boldsymbol{HR-FAR} \right)\boldsymbol{(HR+FAR+}\frac{\boldsymbol{FAR}}{\left( \boldsymbol{1-FAR} \right)}\boldsymbol{)}$ | (5) |
| --- | --- | --- |
|  | $K_{B}=\frac{1}{4}\left( HR-FAR \right) (2-HR+FAR+ \frac{(1-HR)}{HR})$ | (6) |

Importantly, both types of estimates led to equal statistical results. Therefore, only the analysis of the *d’* and *c* estimates are reported below.

**Variation of judgement accuracy across the visual scenery and the eccentricity of the stimuli.**

We also evaluated the participant’s accuracy at discriminating between simultaneous and asynchronous stimuli. A three-way ANOVA evaluated the effect of Context, Eccentricity, and Polar angle on the percentage of correct judgements. The analysis revealed a main effect of the Context (*F*(1,45) = 4.45, *p* = .041, *η^2^_p_* = .09; Fig. S1), such as the participant’s accuracy was significantly lower in the natural (Mean = 60.49, SD = 7.75) compared with the minimal environment (Mean = 62.04, SD = 9.05, Cohen’s *d* = 0.31, CI_95%_ = [0, 0.61]). Also, the analysis showed a significant effect of the Eccentricity *(F*(3,135) = 25.71, *p* = 3.21x 10^-13^, *η^2^_p_* = .364). Planned comparisons showed that participants’ accuracy was lower when the stimuli were presented at 40° rather than 2.5°, 24° and 11.5° of visual angle (two-sided *t*-test _FDR-corrected_, *t*(36) > 4.41, all *p* < .0004, all BF_01_ > 349). Finally, the ANOVA revealed an interaction effect between the Eccentricity and the Polar angle (*F*(9,405) = 4.04, *p* = .0006, *η^2^_p_* = .082), such as the gradual reduction of accuracy with the increase of eccentricity mainly concerned the top and bottom polar angle. The ANOVA reported no other main (*F*(3, 135) = 1.52, *p* = .21) nor interaction (all *F* < 0.62, all *p* > .64) effect. A Bayesian linear model comparison strongly suggests the absence of effect of the Polar angle (BF_01_ = 75, ±1.12%) on the accuracy. In summary, the judgment’s accuracy to the 22 ms asynchrony is reduced in the most natural visual context, but also reduces as the eccentricity of the stimuli increases.


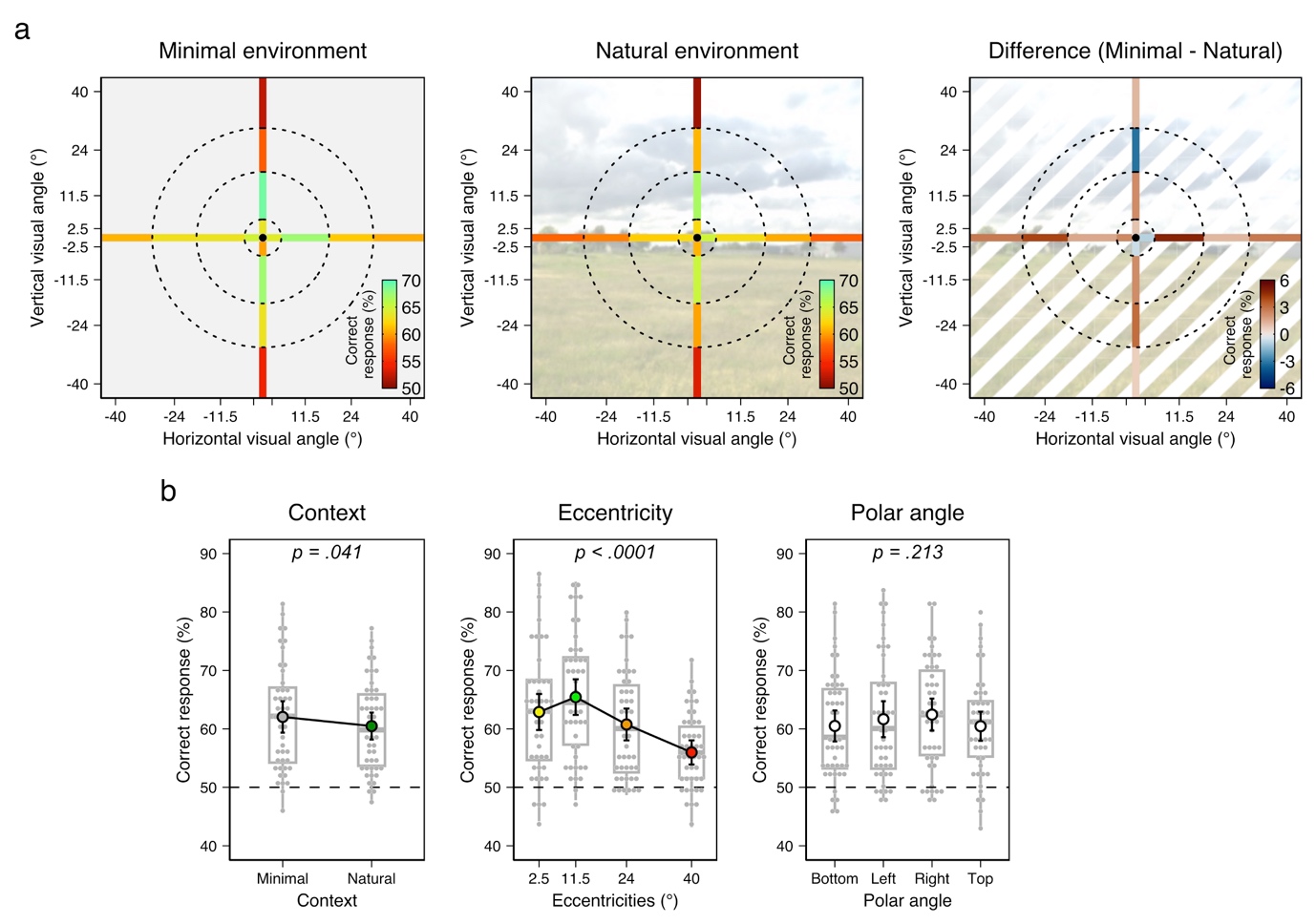


**Fig. S1.** Topographic maps (a) and boxplots (b) of the participants’ accuracy (percentage of correct response) across the visual contexts, the eccentricities of the stimuli and their location in the polar angles. Error bars represent a 95% confidence interval.

**Decision times depends on the visual context and the visual space.**

Finally, we evaluated whether decision times, as part of the decision formation process to judge on the presence or absence of simultaneity, varied across the visual context and the visual space. A three-way ANOVA with the factor Context, Eccentricity, and Polar angle applied to the decision times reported a main effect of both the Context (*F*(1,45) = 5.26, *p* = .027, *η^2^_p_* = .105; Fig. S2) and the Eccentricity (*F*(3,135) = 24.60, *p* = 9.19 x 10^-13^, *η^2^_p_* = .353). In congruence with the main analysis reflecting difficulties at discriminating simultaneous from asynchronous visual stimulation in the natural environment and in the peripheral fields, decision times were longer in the natural scenery and increased with the eccentricity of the stimuli (Cohen’s *d* = 0.34, CI_95%_ = [0.05, 0.62]). Decision times differed between all pairs of eccentricities (two-sided *t*-tests FDR-corrected, all *t*(36) > 2.70, all *p* < .058, all BF_10_ > 3.99). In other words, decision times increase linearly with the eccentricity of the visual stimulation. Furthermore, the ANOVA revealed a main effect of the Polar angle (*F*(3,135) = 17.69, *p* = 9.66 x 10^-10^, *η^2^_p_* = .282), such as decision times were significantly longer when judging the stimuli presented in the top visual field rather than in the other visual fields (two-sided *t*-tests FDR-corrected, all *t*(36) > 4.52, all *p* < 3 x 10^-4^, all BF_10_ > 494). The planned comparisons analysis reported evidence for the absence of difference in decision times between the left, right, and bottom visual fields (all BF_01_ > 5.82). Finally, the ANOVA indicated an interaction effect between the Eccentricity and the Polar angle (*F*(9,405) = 8.33, *p* = 2.03 x 10^-11^, *η^2^_p_* = .156), such as decision times are significantly longer in the case of stimuli presented at 40° of visual angle in the top visual field compared with (1) stimuli presented at other visual angles in the top visual field (all *p* < .002) and (2) stimuli presented at 40° of visual angle in other visual fields (all *p* < .0002). Thus, participants take significantly longer to judge the stimuli presented on the periphery in the top visual field compared with other locations. No other interaction effect was found (all *F* < 1.62, all *p* > .187). This analysis suggests that discriminating simultaneous from asynchronous stimuli requires more time in the naturalistic visual scenery and in peripheral visual fields.


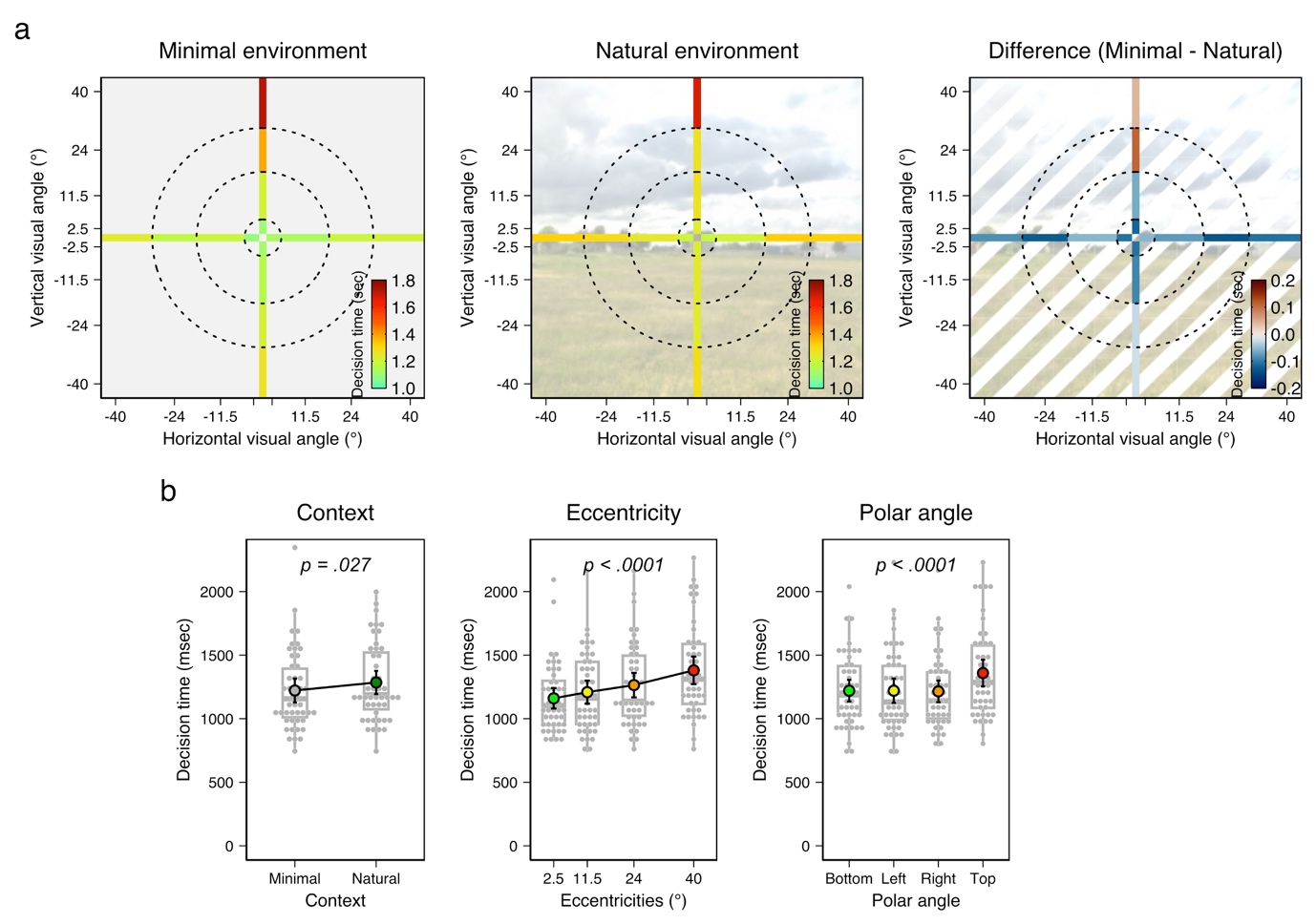


**Fig. S2.** Topographic maps (a) and boxplots (b) of the decision time across visual context, the eccentricities of the stimuli and their location in the polar angles. Error bars represent a 95% confidence interval.

**No response bias across the experimental parameters.**

In a control analysis we verified the participants’ response bias (see Fig. S3). A three-way ANOVA with the factor Context, Eccentricity, and Polar angle applied to the response bias (criterion *c* values) reported no main (all *F* < 3.9, all *p* > .054) nor interaction (all *F* < 1.48, all *p* > .22) effect. Bayesian linear model comparisons strongly suggested the absence of effect of the Context (BF_01_ = 1.62, ±0.77%), the Eccentricity (BF_01_ = 217, ±0.72%) or the Polar angle (BF_01_ = 152, ±0.72%) on the response bias. Altogether, this analysis shows that the probability of reporting the presence or absence of a visual asynchrony independently of the stimulus presentation seem similar across space and visual contexts.


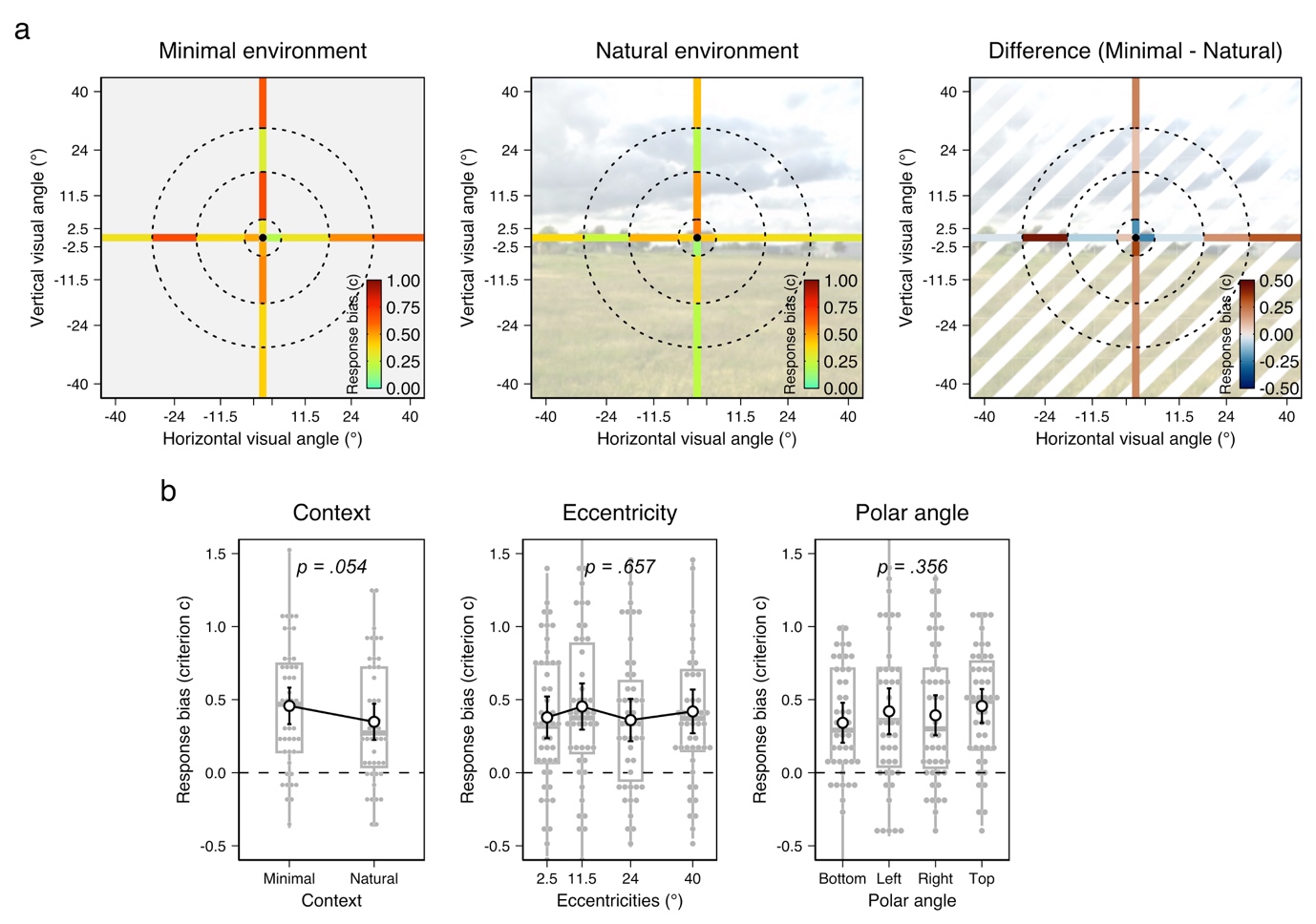


**Fig. S3.** Topographic maps (a) and boxplots (b) of the response bias across the visual contexts, the eccentricities of the stimuli and their location in the polar angles. Error bars represent a 95% confidence interval.

**Fig. S4.** Raw data representing the percentage of ‘asynchronous’ response (a), hit rate (b), and false alarm rate (c) the eccentricities of the stimuli (first row) and their location along the polar angles (second row) for each visual context. Error bars represent a 95% confidence interval.

**Sensitivity to asynchronies across model factors and subscales of the Schizotypal Personality Questionnaire.**

In a control analysis we examined potential relationship between the overall sensitivity to asynchronies and the other factors and subscales of the SPQ. As described in the manuscript, Spearman correlations only report a relationship between the overall sensitivity to asynchronies and the subscale “Odd beliefs or magical thinking” (*r* = -0.31, *p _uncorrected_* = 0.039, BF_10_ = 2.8). P-values did not resist to FDR-corrections for multiple comparisons. This correlation strengthens the link between these results and schizotypy.

**Fig. S5.** Spearman correlations between overall sensitivity to asynchronies and subscales of the SPQ.

**References**

1. Stanislaw, H. & Todorov. Calculation of signal detection theory measures. *Behav. Res. Methods Instrum. Comput.* **3**, 37–149 (1999).

2. Kornbrot, D. E. Signal detection theory, the approach of choice: Model-based and distribution-free measures and evaluation. *Percept. Psychophys.* **68**, 393–414 (2006).
